# The activity of the carbamoyl phosphate synthase 1 promoter in human liver‐derived cells is dependent on hepatocyte nuclear factor 3‐beta

**DOI:** 10.1111/jcmm.13123

**Published:** 2017-03-08

**Authors:** Zhanfei Chen, Nanhong Tang, Xiaoqian Wang, Yanling Chen

**Affiliations:** ^1^ Fujian Institute of Hepatobiliary Surgery Fujian Medical University Union Hospital Fuzhou China; ^2^ Key Laboratory of Ministry of Education for Gastrointestinal Cancer Research Center for Molecular Medicine Fujian Medical University Fuzhou China

**Keywords:** carbamoyl phosphate synthetase 1, promoter, hepatocyte nuclear factor 3‐beta, ammonia detoxification, liver‐derived cell

## Abstract

Carbamoyl phosphate synthase 1 (CPS1) is the rate‐limiting enzyme in the first step of the urea cycle and an indispensable enzyme in the metabolism of human liver. However, CPS1 epigenetic regulation involves promoter analysis and the role of liver‐enriched transcription factors (LETFs), which is not fully elucidated. In this work, the promoter region of hCPS1 gene was cloned, and its activity was investigated. An LETF, hepatocyte nuclear factor 3‐beta (HNF3β), was found to promote the transcriptional expression of CPS1 in liver‐derived cell lines. In addition, dual‐luciferase reporter assay shows that the essential binding sites of the HNF3β may exist in the oligonucleotide −70 nt to +73 nt. Two putative binding sites are available for HNF3β. Mutation analysis results show that the binding site 2 of HNF3β was effective, and the transcriptional activity of CPS1 promoter significantly decreased after mutation. Electrophoretic mobile shift assay (EMSA) and ChIP assay confirmed that HNF3β can interact with the binding site in the CPS1 promoter region of −70 nt to +73 nt promoter region *in vivo* and *in vitro* to regulate the transcription of CPS1. Moreover, HNF3β overexpression enhanced the transcription of CPS1 and consequently improved the mRNA and protein levels of CPS1, whereas the knockdown of HNF3β showed the opposite effects. Finally, urea production in cells was measured, and ammonia detoxification improved significantly in cells after transfection with HNF3β. HNF3β plays a vital role in regulation of CPS1 gene and could promote the metabolism of ammonia by regulating CPS1 expression.

## Introduction

CPS1 is a liver‐specific, intramitochondrial, indispensable enzyme in the metabolism of human liver. CPS1 is the rate‐limiting enzyme in the first step of the catalytic cycle of urea cycle. Previous research showed that CPS1 is at low expression levels in 75% of hepatocellular carcinoma (HCC) 1. Gene mutation and epigenetic regulation may lead to abnormal gene regulation function, and numerous types of cancer cells present abnormal epigenetic patterns. Hypomethylation is a prerequisite for the activation of the DNA proximal promoter region, whereas hypermethylation leads to the silencing of genes 2. Liu 3 speculated that the low CPS1 gene expression in HCC may be related to DNA methylation; in addition, Liu proved that the CpG island hypermethylation of CPS1 gene is a key mechanism for the low CPS1 enzyme expression in the liver cell carcinoma, and two CpG island hypermethylation sites were found in the vicinity of the promoter region. SIRT5, an NAD‐dependent protein deacetylase, plays a pivotal role in the treatment and detoxification of ammonia by activating CPS1 expression 4.

CPS1 is particularly expressed in liver mitochondria and small intestine epithelial cells. CPS1 mutation can cause CPS1 deficiency (CPS1D), which causes hyperammonemia, neonatal death or mental retardation. CPS1D‐associated mutations affect the function of a non‐catalytic 20 kD C‐terminal domain 5. The domain is the allosteric region and combined with *N*‐acetyl glutamate 6; therefore, mutations disrupt the allosteric activation of *N*‐acetyl glutamate on CPS1.

A recent study revealed that the ammonia removal of HepaRG cells through the urea cycle is limited. The low ammonia removal is related to the low expression of the urea cycle enzymes Arg1, OTC, and CPS1 (DMSO presence) 7. Gene regulation of the hepatocyte lineage is mainly at the transcriptional level, and all kinds of LETFs or heterologous nuclear receptors play a vital role 8. However, promoter studies on the low expression of key enzymes in the liver cell urea metabolic pathway are few. CPS1 epigenetic regulation involves promoter analysis, and the role of LETFs in the regulation is not yet fully elucidated. The promoter region of CPS1 gene was cloned, and the key transcription factor, HNF3β, was determined. Furthermore, we found an important HNF3β binding site (−21 nt to −10 nt, relative to the translation initiation site). Our study proved that the HNF3β performs a vital function in the regulation of CPS1 expression.

## Materials and methods

### CPS1 promoter luciferase constructs

Genomic DNA from normal blood cells was extracted by use of Genomic DNA Purification Kit (Promega, Madison, WI, USA) and used as a template for polymerase chain reaction amplification. The plasmid pGL4‐2086 with the CPS1 promoter driving firefly luciferase was constructed by ligation of the PCR‐generated full‐length CPS1 promoter (nucleotides −2086 to +73, relative to the translation initiation site) into the *Xho*I and *Hind*III (Thermo Scientific, Waltham, MA, USA) cleaved sites of the luciferase reporter plasmid pGL4‐Basic (Promega). Various CPS1 promoter deletion constructs, which included pGL4‐1408 (nucleotides −1408 to +73), pGL4‐825 (nucleotides −825 to +73), pGL4‐482 (nucleotides −482 to +73), pGL4‐212 (nucleotides −212 to +73) and pGL4‐70 (nucleotides −70 to +73), were also made by insertion of the corresponding PCR‐generated fragment into the *Xho*Ι and *Hind*III sites of the pGL4‐Basic plasmid. The primers used for amplification are shown in Table 1. The reverse primer −2086/+73R was used together with each of the forward primers.

**Table 1:** List of oligonucleotides used in this study^a^

Oligonucleotides	Sequences (5′ → 3′)	Length of products (bp)
CPS1 promoter cloning		
−2086/+73F	CCG *CTCGAG* CCCAGCCAAAAGTAGGTATTCT	2159
−1408/+73F	CCG *CTCGAG* TCCTAAACTCACCCTCCCG	1481
−825/+73F	CCG *CTCGAG* CTGGGTAGCAGAAGTGAGGAC	898
−482/+73F	CCG *CTCGAG* GCAGTTGATAATACTTTGGGGA	555
−212/+73F	CCG *CTCGAG* TAAAAAGTCAGCCTCACTCTCT	285
−70/+73F	CCG *CTCGAG* TCCCAAACTCTACTCCTAATCC	143
−2086/+73R	CCC *AAGCTT* ATCCAGCTTAAGACTCACCATG	
Site‐directed mutagenesis		
HNF3βmut1F	CCCTCCCCACACCCAGGACTGCTCTTTTAAAATAG	
HNF3βmut1R	CTATTTTAAAAGAGCAGTCCTGGGTGTGGGGAGGG	
HNF3βmut2F	CCAGGTTTGCTCTTTTAAAAAGGTTGCTTTCTTAGGAAATG	
HNF3βmut2R	CATTTCCTAAGAAAGCAACCTTTTTAAAAGAGCAAACCTGG	
EMSA		
Labelled probe F	5′ biotin CTTTTAAAATAGTTGCTTTCTTAGG	
Labelled probe R	5′ biotin CCTAAGAAAGCAACTATTTTAAAAG	
HNF3βmutF	CTTTTAAGGTATCCGCCCTCTTAGG	
HNF3βmutR	CCTAAGAGGGCGGATACCTTAAAAG	
ChIP		
CPS1 ChIP‐F	TCCCAAACTCTACTCCTAATCC	143
CPS1 ChIP‐R	ATCCAGCTTAAGACTCACCATG	
Real‐time RT‐PCR		
CPS1F	TCAAGGCACAGACAGCACAC	
CPS1R	TTCATCCAGAGCAGTAGTATCAGG	
GAPDHF	AGGGCTGCTTTTAACTCTGGT	
GAPDHR	TCTCGCTCCTGGAAGATGGTG	

a Mutant bases are underlined. Restriction sites are shown in italics.

The pGL4‐70 plasmid was used as a template for construction of HNF3β binding sites mutants. The genetailor site‐directed mutagenesis kit (Invitrogen, Carlsbad, CA, USA) was used following the manufacturer's instructions. The primers used for amplification are shown in Table 1. The resulting plasmids were pGL4‐70‐HNF3βmut1 and pGL4‐70‐HNF3βmut2. All constructs were confirmed by DNA sequencing.

### Cell culture and transfection

Human hepatoblastoma cell line HepG2 (HB‐8065, ATCC, Manassas, VA, USA), hepatoma cell line Huh7 (JCRB0403, Japan), embryonic kidney cell line 293A (R705‐07; Invitrogen) and BEL‐7404 (Shanghai Cell Biology Institute of Chinese Academy of Science, Shanghai, China) were maintained in Dulbecco's modified Eagle medium (DMEM; Life Technologies, Grand island, NY, USA) supplemented with 12% (v/v) foetal bovine serum (FBS, Life Technologies). Cells were plated at a density of 2 × 10^5^ cells/well. DNA transfection was carried out in 24‐well plates by use of Lipofectamine 2000 (Invitrogen), in accordance with the manufacturer's recommendations. LETFs for cotransfection were pENTER‐HNF1α, pENTER‐HNF6 and pENTER‐C/EBPβ (Vigene Bioscience, Shandong, China), GV316‐HNF4α and GV230‐C/EBPα (Genechem, Shanghai, China), pFLAG‐HNF3β and pFLAG‐CMV‐2 (donated by Key Laboratory of Ministry of Education for Gastrointestinal Cancer, Fujian Medical University, China).

### Dual‐luciferase reporter assay

Cells were transfected with the CPS1 promoter luciferase reporter constructs. The renilla luciferase expression vector pRL‐TK (Promega) was used for normalization, and the promoterless vector pGL4‐Basic served as the negative control. Cells were lysed 48 hrs after transfection. The Dual‐Luciferase Reporter Assay System (Promega) was used to detect intracellular luciferase activity in 40 μl of cell lysates, following the manufacturers’ recommendations. Luminescence measurement was carried out on an illuminometer (Orion II Microplate Luminometer, Berthold Detection Systems, Germany). Each transfection was performed in triplicate.

### Western blotting

Protein (30 μg) was subjected to 10% sodium dodecyl sulphate–polyacrylamide gel electrophoresis (SDS‐PAGE), and electrophoretic transfer to a polyvinylidene fluoride (PVDF) membrane (Merck Millipore, Darmstadt, Germany). Protein blots were incubated separately with a panel of specific antibodies that included anti‐CPS1 (ab155083; Abcam, Cambridge, UK), anti‐HNF3β (ab108422; Abcam) and anti‐β‐actin (sc‐47778; Santa Cruz Biotechnology, Santa Cruz, CA, USA) antibodies at 1:1000 dilution. Immunoreactivity was detected using a chemiluminescence Western blot immunodetection kit (Invitrogen) according to the manufacturer's instructions and recorded on Hyperfine‐ECL detection film. The amounts of each protein were semi‐quantified as ratios to β‐actin indicated on each gel.

### RNA extraction and real‐time RT‐PCR analysis

Total RNA was extracted from HepG2 cells with TRIzol Reagent (Invitrogen). The reverse transcription reaction was carried out with 3 μg of RNA in a final volume of 20 μl by use of the Transcriptor First Strand cDNA Synthesis Kit (Roche Diagnostics, GmbH, Mannheim, Germany). Quantitative real‐time PCR was performed with the ABI StepOne Real‐Time PCR System (Applied Biosystems, Foster City, CA, USA) and the Fast Start Universal SYBR Green Master Mix (Roche). Glyceraldehyde‐3‐phosphate dehydrogenase (GAPDH) served as an internal control. The primers used for GAPDH and CPS1 amplification are shown in Table 1. Each sample was analysed in triplicate. The relative expression level of CPS1 was calculated by normalization to the endogenous GAPDH mRNA expression prior to comparative analysis by use of the 2^−ΔΔCt^ method as previously described 9. All procedures followed the manufacturer's instructions.

### Nuclear extraction and EMSA

The 5′‐biotin end‐labelled oligonucleotides that corresponded with the HNF3β transcription factor recognition sequences within the CPS1 promoter region were synthesized (Viagene, Jiangsu, China) and used as probes. Unlabelled oligonucleotides, either wild‐type (cold probes) or mutated (mutated cold probes), were used as competitors. Oligonucleotides sequences are shown in Table 1. A Nuclear and Cytoplasmic Protein Extraction Kit (Beyotime, Jiangsu, China) was used to prepare nuclear proteins from HepG2 cells. An EMSA Gel Shift Kit (Viagene) was used to examine the interaction between HNF3β in the nuclear extract with the probe, in accordance with the manufacturer's instructions. Briefly, 20 μg of nuclear extracts were incubated with 0.5 μg of poly (dI‐dC) and 13.8 nM labelled probe in a final volume of 18 μl. The competition assay was performed using 50‐ to 100‐fold molar excess of cold probes or cold mutated probes preincubated with the reaction mixture before addition of biotin‐labelled probes. In supershift assays, 4 μg of the specific antibodies (anti‐HNF3β) was added to the mixtures of nuclear extracts and DNA probes (In here, the quality of poly (dI‐dC) we use is 0.8 μg). The DNA–protein complexes were incubated at 25°C for 20 min., then subjected to electrophoresis on a 6.0% non‐denaturing pre‐made polyacrylamide gel (Invitrogen). Next, the complexes were transferred to a nylon Binding‐membrane (Viagene) and fixed nearly 10 cm below the UV lamp for 30 min. cross‐linking. The biotin end‐labelled DNA was detected by addition of streptavidin‐horseradish peroxidase conjugate and chemiluminescent substrate.

### Chromatin immunoprecipitation assay (ChIP)

HepG2 cells (10^7^) were cultured and cross‐linked by adding formaldehyde to a final concentration of 1% at room temperature for 10 min. The cross‐linking was stopped by addition of 125 mM glycine at room temperature for 5 min. Cells were rinsed twice and collected with ice‐cold phosphate‐buffered saline (PBS), then resuspended in 0.75 ml FA Lysis Buffer (Abcam, Cambridge, MA, USA) containing a protease inhibitor cocktail (Roche) and incubated on ice for 10 min. Cell lysates were sonicated for 20 min. with a Branson model 350 Sonifier (Branson Sonic Power Co., Danbury, CT, USA) at 20% duty cycle. This conditions yielded DNA fragments ranging from 200 to 500 base pair (bp) in size. Before immunoprecipitation, 50 μl of each lysate was removed for analysis of input chromatin DNA. Immuoprecipitation was conducted with 5 μg of specific antibodies (anti‐HNF3β) followed by 80 μl of pre‐blocked protein A/G. The normal rabbit IgG or pre‐blocked protein A/G (no antibody control) was also performed for control purpose. Following elution and proteinase K digestion, DNA was recovered by phenol/chloroform extraction and ethanol precipitation in the presence of glycogen and dissolved in 100 μl distilled sterile water. Bound target DNA fractions were analysed by PCR. The primers used are shown in Table 1, which amplified a region that spanned nucleotides −70 to +73 (containing the HNF3β binding sites) of the CPS1 promoter. The resulting PCR products were electrophoresed on 2% agarose gels and stained with ethidium bromide.

### RNA interference assay

The small interfering RNA (siRNA) targets for HNF3β (5′‐GCCGUCCGACUGGAGCAGCUACUAUdTdT‐3′) were described by Tomaru 10. All siRNA duplexes, which included a negative control that had no homology with known human genes, were synthesized chemically by the GenePharma Company (Shanghai, China); 100 pmol of HNF3β‐siRNA or the negative control was used for transfection. Total RNA and proteins were isolated at 48 hrs after transfection in accordance with the manufacturer's instructions.

### Identification of putative transcription factor binding sites

A computer‐based search for potential transcription factor binding site motifs was carried out on the TRANSFAC 6.0 (transfac.gbf.de/) professional database by use of TFSEARCH (http://www.cbrc.jp/research/db/TFSEARCH.html) 11, 12, TESS (http://www.cbil.upenn.edu/cgi-bin/tess/tess) programs 13 and TFbind (http://tfbind.hgc.jp/) 14.

### Measurement of Urea production in cells

1 × 10^5^ of cells were resuspended in 100 μl growth medium and added to a 96‐well culture plate. Cells were transfected transiently with pFLAG‐HNF3β and pFLAG‐CMV‐2 (control), respectively, when the cell density was about 80% and incubated at 37°C for 12 hrs, then the supernatant was replaced by 100 μl growth medium or STD buffer with 0–40 mM NH_4_Cl. After another 12 hrs, 50 μl supernatant was used for the detection assay. Twenty‐five microlitres of 1:3 sulphuric acid/phosphoric acid (by volume) was added, and urea production was determined by adding 25 μl α‐isonitrosopropiophenone (9% in absolute ethanol), incubating at 100°C in the dark for 15 min., and measuring absorbance at 490 nm. Meanwhile, the standard curve and regression equation for detection were produced using a series of urea standards with different concentration, which were used to calculate the urea concentration in the samples 15.

### Statistical analysis

Statistical analyses were performed with SPSS software (SPSS13.0 version for windows) (SPSS Inc., Chicago, IL, USA) by use of analysis of variance (anova) and *t*‐test. All values were expressed as means ± standard deviation (SD) from duplicate experiments. A *P*‐value <0.05 was considered to be statistically significant.

## Result

### Positive regulation of CPS1 promoter transcription by HNF3β

**Figure 1:**
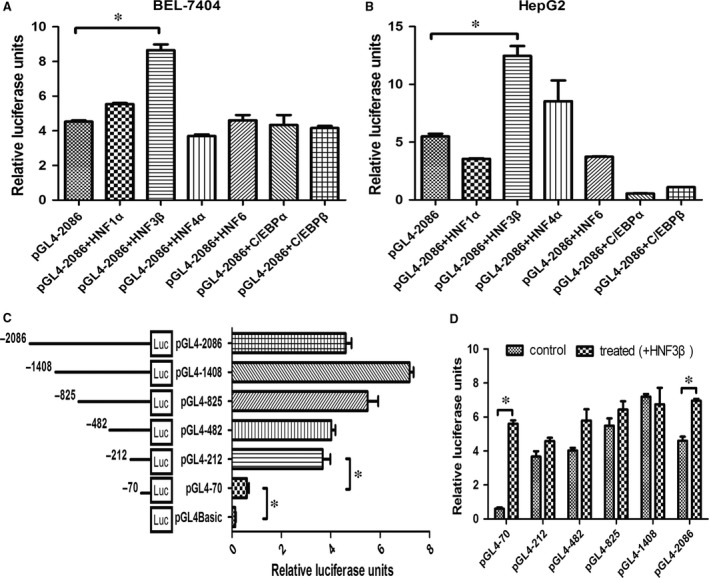
The regulatory effect of HNF3β on CPS1 promoter transcription. (**A**) and (**B**), The plasmid pGL4‐2086 (1 μg) was transfected transiently with LETFs (HNF1α, HNF3β, HNF4α, HNF6, C/EBPα and C/EBPβ) (500 ng), respectively, into the HepG2 and BEL‐7404 cells. Luciferase activities were measured 48 hrs after transfection, and the plasmid pGL4‐2086 served as the negative control. (**C**), Activity analysis of CPS1 promoter. BEL‐7404 cells were transfected with 1 μg each of the CPS1 promoter constructs (reporter plasmid); 100 ng of the renilla luciferase expression vector pRL‐TK was used for normalization, and the promoterless vector pGL4Basic served as the negative control. Luciferase activities were measured 48 hrs after transfection. (**D**), 1 μg each of the CPS1 promoter constructs was cotransfected with 500 ng HNF3β, respectively, into the BEL‐7404 cells. In each recombinant plasmid, fluorescent activity of non transfected with HNF3β served as the negative control. Each transfection was performed in duplicate, and the data were expressed as the mean ± SD of three separate experiments (*P < 0.05).

The LETF family includes hepatocyte nuclear factor (HNF)‐1, (HNF)‐3, (HNF)‐4 and (HNF)‐6, CCAAT/enhancer binding protein (C/EBP), D site‐binding protein (DBP) and CREB‐H. LETFs play an important role in the regulation of hepatic cell lineage genes. Full‐length constructs of the CPS1 gene promoter (pGL4‐2086) were transfected transiently and cotransfected with the transcription factors HNF1α, HNF3β, HNF4α, HNF6, C/EBPα and C/EBPβ, respectively, into HepG2 and BEL‐7404 cells. As shown in Figure 1A to 1B, the CPS1 transcription activity of cotransfected HNF3β is significantly higher than that of HNF1α, HNF4α, HNF6, C/EBPα and C/EBPβ in the HepG2 and BEL‐7404 cells. HNF3β promotes the transcriptional regulation of CPS1 promoter in liver‐derived cells.

Next, the full length and a series of 5′‐deletion constructs of the CPS1 gene promoter (pGL4‐1408, pGL4‐825, pGL4‐482, pGL4‐212 and pGL4‐70) were transfected transiently into BEL‐7404 cells. The plasmid pGL4‐212 show full maximum luciferase activity relative to the full promoter region (Fig. 1C), suggesting that nucleotides −212 nt to +73 nt is crucial for the full activity of the CPS1 promoter. In addition, all recombinant promoter fragments were, respectively, cotransfected with HNF3β, demonstrating that the fluorescence activity of pGL4‐70 was significantly higher than that of transient transfection after cotransfection with HNF3β (Fig. 1D). The experimental data indicate that the essential binding sites of the HNF3β may exist in the oligonucleotide −70 nt to +73 nt; therefore, the recombinant plasmid pGL4‐70 was used for further studies.

### Identification of HNF3β binding sites in −70 nt to +73 nt region of the CPS1 promoter

**Figure 2:**
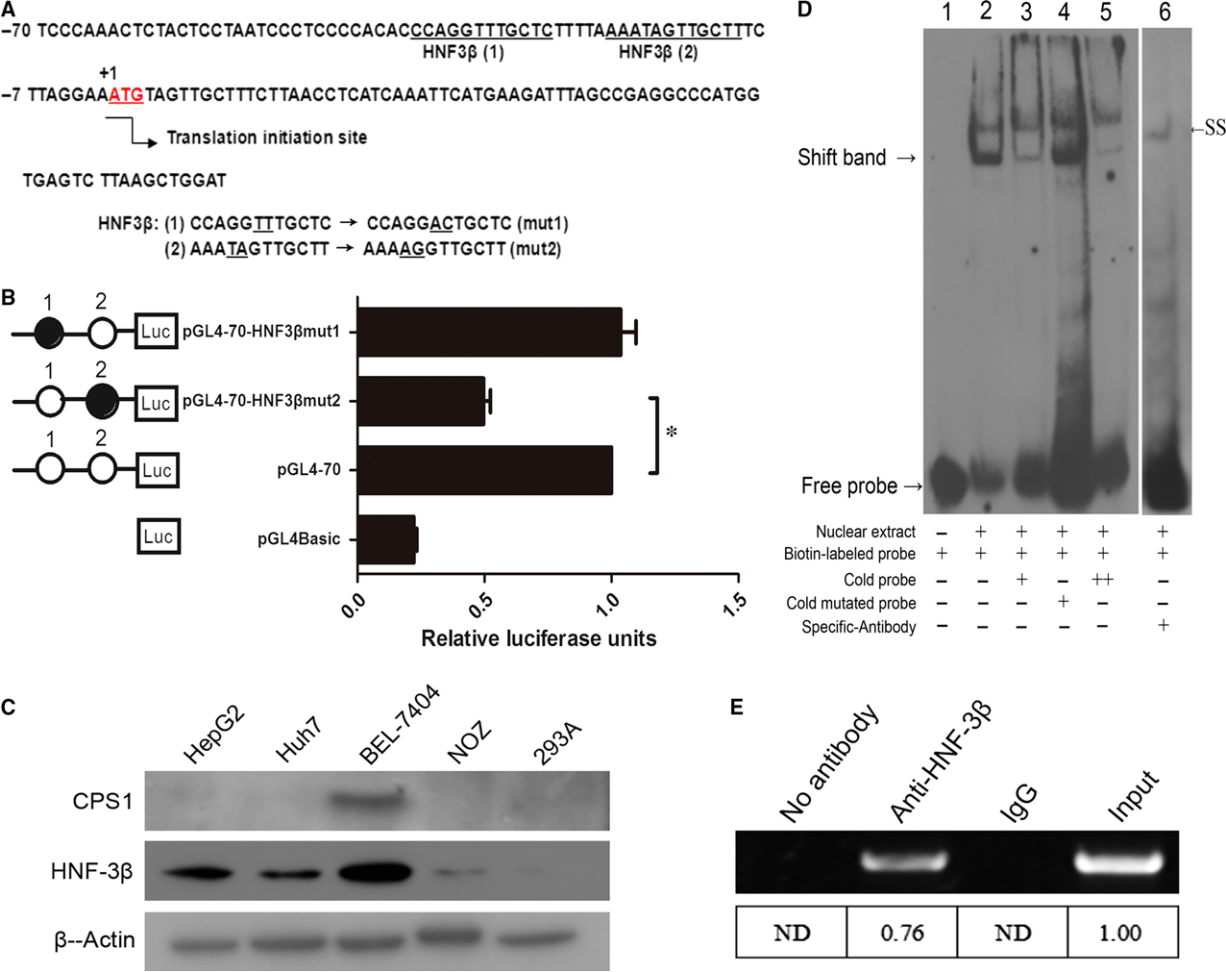
Binding sites of HNF3β in the CPS1 promoter. (**A**), Nucleotide sequence of −70 nt to +73 nt region of CPS1 promoter is shown. The translation initiation site (+1) is indicated by the arrow. The putative HNF3β binding sites in the nucleotide region −38 nt~−27 nt (site 1) and −21 nt~−10 nt (site 2) are underlined. In the binding site 1, TT is mutation into AC, named mut1. Similarly, TA is mutation into AG named mut2 in the binding site 2. (**B**), Effect of mutation of the HNF3β binding sites on the activity of the CPS1 promoter. HNF3β binding sites were subjected to site‐directed mutagenesis. A total of 1 μg of mutants together with 100 ng of pRL‐TK was cotransfected into HepG2 cells. pGL4Basic served as the negative control. Luciferase activities were measured 48 hrs after transfection. The relative luciferase units (RLU) were obtained (right) by comparison with the wild‐type of pGL4‐70, which was set to 1. (**C**), Western blot analysis of CPS1 and HNF3β expression in the various cell lines; 30 μg of cellular proteins was used in the Western blot. β‐Actin serves as an internal control. (**D**), EMSA of HNF3β. Competition assays with unlabelled cold probe at a concentration of 50 (lane 3)‐ or 100 (lane 5)‐fold molar excess over that of the biotin‐labelled probe (lane 2). In the lane 6, supershift assays were carried out with 4 μg specific antibody raised against HNF3β and the quality of poly (dI‐dC) is 0.8 μg. An arrowhead indicates the shift band (SB, left) and the supershift band (SS, right). (**E**), ChIP assay. Chromatin from HepG2 cells was immunoprecipitated with the anti‐HNF3β. The total extracted DNA (Input DNA, 5%) prior to immunoprecipitation and the immunoprecipitated samples were PCR‐amplified using primers specific to a region that spanned −70 nt to +73 nt (containing the HNF3β binding sites) of the CPS1 promoter. The normal rabbit IgG or no antibody control was also performed for control purpose. Band signals were quantified using the densitometric software, and the relative intensities to the input which was set to 1.00 were calculated. ND: None detected (*P < 0.05).

TFSEARCH, TESS and TFBIND software were used to analyse the potential HNF3β binding sites in the nucleotides −70 nt to +73 nt, and a comprehensive evaluation of the optimal two binding sites 1 and 2 (Fig. 2A) were selected for subsequent experiments. Then, site‐directed mutants of the putative HNF3β binding sites were generated and named pGL4‐70‐HNF3βmut1 and pGL4‐70‐HNF3βmut2, and the promoter activities of the corresponding constructs were measured. As shown in Figure 2B, mutations in HNF3β binding site 2 (pGL4‐70‐HNF3βmut2) led to a reduction in the promoter activity of approximately 52% to 57% compared with the control non‐mutated construct (pGL4‐70), not HNF3β binding site 1 (pGL4‐70‐HNF3βmut1). The results show that the binding site 2 of the transcription factor HNF3β was effective, and the transcriptional activity of the CPS1 promoter was significantly decreased after mutation. The CPS1 promoter displays the highest activities in liver‐derived cell lines; therefore, we speculate that the promoter may be closely related to the high protein levels of endogenous HNF3β. As shown in Figure 2C, the HNF3β and CPS1 protein were measured in various cell lines. The protein levels of HNF3β in liver‐derived cell lines HepG2, Huh7 and BEL‐7404 were higher than those in the other cell lines. However, CPS1 protein was not detectable in HepG2 and Huh7 cells. This result is consistent with the study of Butler 16 and conforms to the view of Siddiqui 1, who demonstrated that the low level of CPS1 is expressed in 75% of human HCC tissues.

The role of HNF3β binding site was further verified by EMSA. The results demonstrate that the nuclear extract could ‘shift’ the position of the biotin‐labelled probe because of a change in electrophoretic mobility caused by dsDNA/protein complex formation (Fig. 2D, lane 2); moreover, the DNA‐transcription factor complexes could be ‘super‐shifted’ by the addition of antibodies directed against human HNF3β (Fig. 2D, lane 6). In addition, a competition assay showed that preincubation with 50‐ or 100‐fold molar excess of cold probe (Fig. 2D, lane 3 and lane 5), but not the cold mutated probes (Fig. 2D, lane 4), evidently diminished the intensity of the bands.

To determine whether the HNF3β is associated with CPS1 promoter *in vivo*, we performed ChIP assay with specific antibody and polymerase chain reaction (PCR) using the primers against the regulatory elements of the CPS1 promoter. The results show that a 143‐bp DNA fragment covering HNF3β was amplified by chromatin immunoprecipitation with an anti‐HNF3β antibody (Fig. 2E). The same band was obtained with the input DNA, whereas the normal IgG control and no antibody control did not result in immunoprecipitation of DNA fragments detectable by PCR amplification.

Taken together, our results confirm that HNF3β can interact with the binding site in the CPS1 promoter region of −70 nt to +73 nt promoter region *in vivo* and *in vitro* to regulate the transcriptional regulation of the CPS1 promoter.

### Overexpression of HNF3β and interference of HNF3β siRNA

**Figure 3:**
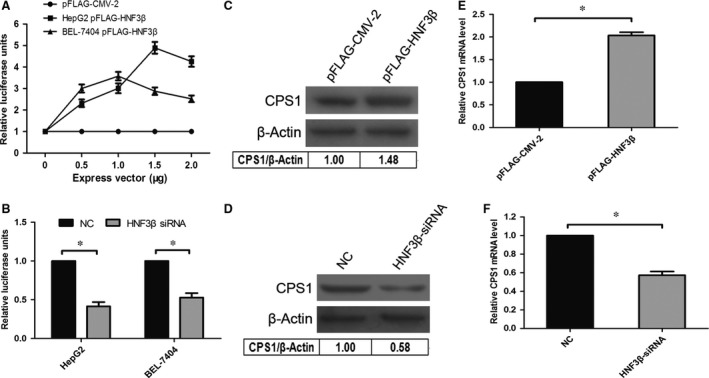
Transcription regulation of CPS1 promoter by HNF3β. (**A**), Overexpression of HNF3β enhances CPS1 promoter activities. HepG2 and BEL‐7404 cells were cotransfected with 0.2 μg of pGL4‐70 and increasing amounts (0, 0.5, 1, 1.5 or 2.0 μg) of expression vectors (pFLAG‐HNF3β or empty vector pFLAG‐CMV‐2); 10 ng of pRL‐TK was used to normalize the transfection efficiency. Cells were harvested 48 hrs after transfection. The relative luciferase units (RLU) were obtained by comparison with the pFLAG‐CMV‐2, which was set to 1. Each transfection was performed in duplicate, and the data were expressed as the mean ± SD of three separate experiments. (**B**), Knockdown of endogenous HNF3β decreased CPS1 promoter activity. HepG2 and BEL‐7404 cells were cotransfected with HNF3β siRNA and 0.2 μg of pGL4‐70. At 48 hrs after transfection, the relative luciferase units (RLU) were obtained by comparison with the negative control (NC), which was set to 1. Each transfection was performed in duplicate, and the data were expressed as the mean ± SD of three separate experiments. *P < 0.05 *versus* NC. (C) and (D), Western blot analysis in BEL‐7404 cells after overexpression of HNF3β or interference of HNF3β siRNA. (C): BEL‐7404 cells were transfected with 0.5 μg of pFLAG‐HNF3β or pFLAG‐CMV‐2 (empty vector); (D): BEL‐7404 cells were treated with 100 pmol of HNF3β siRNA or negative control (NC). Cells were harvested 48 hrs after transfection; 30 μg of cellular proteins was used in the Western blot and β‐actin served as a loading control. (E) and (F), Influences of HNF3β or HNF3β siRNA on CPS1 gene mRNA level in HepG2 cells. Overexpression of HNF3β increased CPS1 gene transcription, HepG2 cells were transfected with 0.5 μg of pFLAG‐HNF3β or empty control pFLAG‐CMV‐2 (E); knockdown of endogenous HNF3β decreased CPS1 gene transcription, HepG2 cells were treated with 100 pmol of HNF3β siRNA or negative control (NC) (F). Cells were harvested 48 hrs after transfection, 3 μg of the total RNA was used to detect the CPS1 mRNA level by real‐time RT‐PCR (*P < 0.05).

To elucidate the transcriptional regulation of HNF3β on the CPS1 promoter, we cotransfected pGL4‐70 with different doses of pFLAG‐HNF3β and HNF3β siRNA in HepG2 and BEL‐7404 cells. As shown in Fig. 3A and 3B, the fluorescence activity of CPS1 promoter increased with pFLAG‐HNF3β overexpression, which was dependent on HNF3β concentration (Fig. 3A). Meanwhile, the fluorescence intensity of cotransfected HNF3β siRNA was significantly lower than that of the negative control (Fig. 3B). In addition, the levels of CPS1 in protein (BEL‐7404 cell) and mRNA (HepG2 cell) also show the same trend (Fig. 3C–3F). The results show that the overexpression of pFLAG‐HNF3β promotes the transcription of the CPS1 promoter and then increases the level of CPS1 mRNA (Fig. 3E), ultimately resulting in the enhancement of CPS1 protein level (Fig. 3C). On the contrary, the interference of HNF3β siRNA reduced the transcription of the CPS1 promoter and inhibited the expression of CPS1 mRNA (Fig. 3F), and the expression of CPS1 protein level also decreased (Fig. 3D).

### Comparison of ammonia detoxification by cells

**Figure 4:**
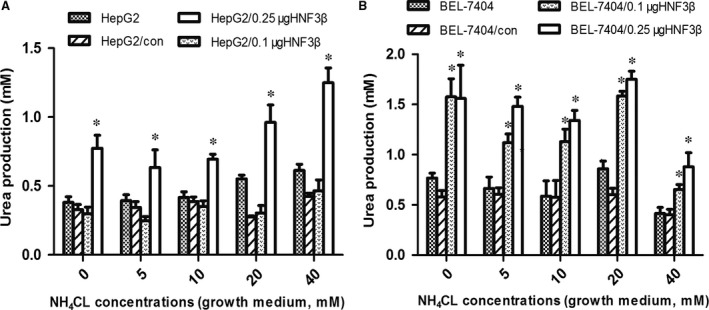
Urea production in cells with different concentrations of ammonia. With increasing ammonia concentration, (**A**) urea production in HepG2 and HepG2/0.25μgHNF3β cells increased. *denotes a significant difference compared to HepG2 (*P* < 0.05, *n* = 3). (**B**) While in BEL‐7404/0.1μgHNF3β and BEL‐7404/0.25μgHNF3β cells, urea production increased at the beginning and decreased later. The median lethal dose (LD50) of NH4Cl in each cell line was different. The cell viability and function gradually declined with increasing ammonia concentration; thus, the amount of urea products decreased. *denotes a significant difference compared to BEL‐7404 cells (*P* < 0.05, *n* = 3).

The urea cycle is one of the metabolic pathways of ammonia. Thus, the production of urea is used to evaluate the capacity for ammonia detoxification in cells. Urea synthesis in HepG2 and BEL‐7404 cells is shown in Fig. 4A and 4B. Urea products increased in different degrees with increasing ammonia concentration through transfection with pFLAG‐HNF3β, although urea production in cells may be reduced after transient transfection with pFLAG‐CMV‐2 (control). The transcription factor HNF3β could promote the metabolism of ammonia by regulating the expression of CPS1.

## Discussion

We demonstrated the relationship between LETFs and CPS1 expression in liver‐derived cell lines. Our data showed that HNF3β played an important role in the regulation of CPS1 gene, and a potent HNF3β binding site in CPS1 promoter was found.

The transcription of gene is tissue‐specific and is mainly controlled by the interaction between transcription factors and their corresponding *cis*‐acting elements 17, 18. After the online software prediction of the existence of LETF binding sites in −2086 nt to +73 nt, we selected the HNF1α, HNF3β, HNF4α, HNF6, C/EBPα and C/EBPβ as the research object. Our study shows that the activity of CPS1 significantly enhanced after cotransfection with HNF3β, whereas cotransfection with C/EBPα and C/EBPβ does not enhance transcription. Our findings are not consistent with the study of Howell 19 and Lagace 20 that multiple C/EBP binding sequences were found in the promoter of rat CPS1. A minimum of two possibilities could explain this paradox. First, other transcription factors may do the opposite of C/EBP in the human CPS1 promoter. However, the more plausible explanation is that despite the function of the C/EBP binding site in the rat CPS1 promoter, whether this function is true in humans was unknown. We performed luciferase activity on all truncated fragments, and transient cotransfection with C/EBPβ was not enhanced (data not shown). The exact mechanism needs further study. In addition, the −212 nt to +73 nt sequence retains full maximum luciferase activity of the CPS1 promoter, thereby indicating that the region is the core promoter region of the CPS1 gene. Moreover, we identified two potential HNF3β binding sites (−38 nt to −27 nt and −21 nt to −10 nt) in the −70 nt to +73 nt region. However, due to the limitations of the prediction software, HNF3β binding sites are not shown in −212 nt to −70 nt. We speculate that other important transcription factors may be involved.

The important role that HNF3β plays in the regulation of CPS1 expression is supported by the following experiments. First, site‐directed mutagenesis was used to construct the mutant recombinant plasmid according to the prediction of the binding site, and binding site 2 was found to exert a definite effect on the transcription of CPS1. Second, probes were synthesized on the basis of the binding site to carry out EMSA and ChIP assay by use of HNF3β specific antibody, confirming that HNF3β can interact with the CPS1 promoter *in vivo* and *in vitro*. In addition, the overexpression of HNF3β and interference of HNF3β‐siRNA strengthened the conclusion that the transcription of CPS1 gene is regulated by HNF3β. The overexpression of HNF3β promotes the transcription of CPS1 and then increases the level of CPS1 mRNA and protein expression. Conversely, the expression levels of CPS1 mRNA and protein are significantly reduced after HNF3β interference. Finally, urea production in HepG2 and HepG2/HNF3β cells was measured, and ammonia detoxification was improved more significantly in HepG2/HNF3β cells compared with HepG2 cells.

The HNF3 gene family consists of HNF3α, HNF3β and HNF3γ, and the family structure is characterized by a helix/fork DNA binding region containing approximately 110 amino acids. This area is required for nuclear localization and transcriptional activation, which can identify the same DNA sequence. The area shows a high degree of continuity in the evolution and existence of their family members from yeast to humans 21. The HNF3 gene family is involved in important metabolic and detoxification processes. HNF3α functions in regulating the activation of glucagon. The expressions of tyrosine aminotransferase and phosphoenolpyruvate carboxykinase were significantly reduced in the liver cells of HNF3γ gene deletion, and the serum glucose level was decreased, as well 22. The excessive expression of HNF3β may lead to the decrease in glycogen synthesis ability 23. Therefore, the HNF3 binding site in the promoter of CPS1 was associated with the regulation of serum glucose.

CPS1 gene expression was closely related to the glucocorticoid and glucagon. The CPS1 distal enhancer element (GRU 24, 25), proximal enhancer element (GAGA‐box 26, GRE 27) and the core promoter region are indispensable for the glucocorticoid cascade response element. CREB stimulates the transcription of CPS1 and ASS based on glucagon signals 28, 29. The transcription of partial enzymes in the urea cycle is also regulated by glucocorticoid and the glucagon signalling pathway, but the regulation mechanism is different 30, 31, 32.

In summary, CPS1 gene is regulated by the transcriptional regulation of the LETF HNF3β. CPS1 gene expression is closely linked with numerous kinds of liver diseases. We hope that the research on the CPS1 transcription presents a certain guiding significance for the treatment of disease. Odom 33 proposed that a large number of liver‐specific transcription factors binding to the promoter indicated a high possibility of expression of the gene in liver. Moreover, a positive correlation was shown between the number of transcription factors binding to the promoter and the level of gene expression. The regulation of liver‐specific gene is more likely to be accomplished by a number of LETFs and heterologous nuclear receptors, and a single LETF or heterologous nuclear receptor cannot correct the complex intracellular regulation. Therefore, further studies are needed to elucidate other potential *trans*‐acting factors interacting with CPS1 gene, as well as the regulation mechanism of their common components. Finally, this work will provide appropriate intervention targets for our study on constructing novel liver cells with a better solution to address ammonia toxicity.

## Conflict of interest

The authors confirm that there are no conflict of interests.
